# Load Measurement of the Cervical Vertebra C7 and the Head of Passengers of a Car While Driving across Uneven Terrain

**DOI:** 10.3390/s21113849

**Published:** 2021-06-02

**Authors:** Martin Svoboda, Milan Chalupa, Karel Jelen, František Lopot, Petr Kubový, Milan Sapieta, Zdeněk Krobot, Marcin Suszyński

**Affiliations:** 1 Faculty of Mechanical Engineering, Jan Evangelista Purkyně University, 400 96 Ústí nad Labem, Czech Republic; 2 Faculty of Military Technology, University of Defence, 662 10 Brno, Czech Republic; milan.chalupa@unob.cz (M.C.); zdenek.krobot@unob.cz (Z.K.); 3 Faculty of Physical Education and Sport, Charles University, José Mártího 31, 162 52 Praha 6, Czech Republic; jelen@ftvs.cuni.cz (K.J.); lopot@ftvs.cuni.cz (F.L.); kubovy.petr@seznam.cz (P.K.); 4 Faculty of Mechanical Engineering, University of Žilina, 010 26 Žilina, Slovakia; milan.sapieta@fstroj.uniza.sk; 5 Faculty of Mechanical Engineering, Poznan University of Technology, 60-965 Poznan, Poland; marcin.suszynski@put.poznan.pl

**Keywords:** measurement, dynamic load, car, experiment, human

## Abstract

The article deals with the measurement of dynamic effects that are transmitted to the driver (passenger) when driving in a car over obstacles. The measurements were performed in a real environment on a defined track at different driving speeds and different distributions of obstacles on the road. The reaction of the human organism, respectively the load of the cervical vertebrae and the heads of the driver and passenger, was measured. Experimental measurements were performed for different variants of driving conditions on a 28-year-old and healthy man. The measurement’s main objective was to determine the acceleration values of the seats in the vehicle in the vertical movement of parts of the vehicle cabin and to determine the dynamic effects that are transmitted to the driver and passenger in a car when driving over obstacles. The measurements were performed in a real environment on a defined track at various driving speeds and diverse distributions of obstacles on the road. The acceleration values on the vehicle’s axles and the structure of the driver’s and front passenger’s seats, under the buttocks, at the top of the head (Vertex Parietal Bone) and the C7 cervical vertebra (Vertebra Cervicales), were measured. The result of the experiment was to determine the maximum magnitudes of acceleration in the vertical direction on the body of the driver and the passenger of the vehicle when passing a passenger vehicle over obstacles. The analysis of the experiment’s results is the basis for determining the future direction of the research.

## 1. Introduction

There is a constant dynamic load of the movement apparatus in a person’s everyday life. The well-being of a person often deteriorates over time, mostly when it comes to prolonged unilateral activities. Because of the excessive load during work activities there are often problems such as backache, degeneration of cervical vertebrae, so-called ‘tennis elbows’ or even the gangrene of fingers or an illness leading to death. Some of the most affected groups are truck drivers and train operators exposed to vibrations while moving in a wheel or rail-based vehicles. Other professionals who often encounter these conditions are sportspeople, construction workers, workers using sledgehammers or jackhammers, sedentary job workers, etc. [1,2].

In the case of cars, the issue of the comfort of the vehicle driver and other passengers is often addressed, and currently solved in line with the driveability of vehicles and especially their driving safety [3,4].

Properly set driving comfort will cause the least possible nerve and muscle fatigue, and it is mainly affected by mechanical shocks, noise, visibility, thermal comfort and a number of other factors. Comfort for the driver and passengers is largely ensured by the car’s interior, which provides them with driving comfort, protects from by unwanted dynamic loads or noise and allows lightness and ease when getting on and off [1].

The degree of overload of the organism can manifest itself in humans in the form of headaches or pain in the cervical or lumbar spine. Many studies deal with the long-term burden on the body of drivers or other workers [5].

At the beginning of 1970, biomechanics had already taken an interest in body damage as a result of external mechanical causes. It mainly dealt with injuries caused by road accidents and sports injuries. There was an effort to increase the prevention of these accidents. The main focus was directed toward the most common causes of serious injuries of the head, chest and spine [6].

Vertebrogenic disorders are a common health problem today. “About 60% of patients coming to the doctor in the Czech Republic report back pain” (Dylevský 2009). There are several reasons for these problems. They can be caused by hypokinesia (lack of exercise) or, conversely, by hyperkinesis (an excessive amount of exercise or excessive exercise). Specific causes include unhealthy lifestyles and associated lack of exercise, obesity, sedentary employment, etc. Conversely, back pain is caused in many cases by employment which by its very nature places disproportionate demands on the spinal system. This applies, for example, to professional drivers and workers with heavy mechanical machines. Last but not least, back pain caused by hyperkinesia can be traced in professional athletes [1,3,4,7].

Many studies (e.g., Rubin et al. 2008, Jandák 2007, Fritz 2000) mention that long-term body exposure to vibrations leads to an unfavorable response in the human body, which can result in irreversible damage. People are exposed to these kinds of vibrations, for instance, while using vehicles and working with mechanical machines in machinery, metallurgical industry, construction or mining industry [3,4,7].

The aim of the study (Bovenzi et al. 2002) was to measure car seat vibrations in 12 taxis in operation under current working conditions. The results were evaluated according to the health norms ISO 2631-1:1997. The relationship between the total traveled distance and whole-body vibrations was reviewed. The whole-body vibrations were measured for over four hours on a route around Fukuoka City, in Japan, under normal conditions. The roads in this region are paved, without significant inequalities. Thus the researchers estimated that the road conditions for taxis would be similar. The study’s outcome was that 83% of taxi drivers fall within ‘the potential risk’ zone [8].

A study (Kumar et al. 1999) examined the effect of whole-body vibrations on the lumbar spine of farmers driving a tractor. There were two test groups: the first one consisted of 50 farmers who regularly use a tractor (they are commonly exposed to the vibrations transmitted to the human body); the other group consisted of 50 farmers who do not use a tractor at all. They were divided by age, gender, economy and everyday labor. Both groups underwent magnetic resonance (MRI) in order to assess the effect of these vibrations on degenerative changes in the back. The researchers also measured the magnitude of vibrations emitted by tractors. The results of the study showed that a backache was more common in the group of tractor drivers (40%) than in the farmers who did not use these vehicles (18%) [9].

The focus of a study by Israeli researchers (Alperowitch-Najenson et al. 2010) was to find out about the prevalence of LBP (low back pain) among Israeli professional bus drivers and to evaluate the association of LBP with psychosocial and ergonomic risk factors. This cross-cutting study was carried out with the help of the bus drivers of the biggest public transport company in the Metropolitan region of Tel Aviv [10]. The result, a 45% LBP prevalence in Israeli drivers, is comparable with other study results (Robb, Mansfield 2007), where a 60% prevalence of LBP was discovered (in a 12-month period) among professional truck drivers. Furthermore, there was a different study on taxi drivers in Taipei, where the discovered LBP prevalence was 51% (also in a 12-month period) [11].

In another work (Netterstrom and Juel 1989), the LBP occurrence among public bus drivers in Denmark was assessed, and a 57% LBP prevalence was discovered. In the following study (Magnusson et al. 1996), a group of American and Swedish bus drivers was tested, and a 60% prevalence of LBP was reported. These problems required 18 days of sick leave on average. When compared, the truck drivers’ group suffers from backache more often than the group of workers with sedentary jobs [12].

A further study (Donnelly et al. 2009) proved that the car seat’s ability to adjust to the driver is associated with the onset and range of the driver’s discomfort. The use of a lumbar support system proved to be effective in keeping the physiological lordotic position of the spine while sitting, which was linked to decreasing discomfort in the lumbar region. This intervention also proved that discomfort is reduced by increasing or maintaining local tissue nutrition [13].

In yet another study (Chen et al. 2005), the researchers took an interest in LBP occurrence among taxi drivers. This cross-cutting study aimed to research the influence of the seat angle, using the lumbar rest, and the prevalence of clinically significant LBP among taxi drivers. With 224 test subjects, the average angle value of the back/thigh was 80.6°. Fifty-five per cent of taxi drivers used the lumbar rest regularly; however, 25% reported significant LBP. LBP prevalence was 23%, 37% and 9% among the drivers who had the back/thigh angle < 86°, 86–91° and > 91° on average, respectively. The drivers who did not use a lumbar rest had an 18% LBP prevalence compared to 34% with adjusted rest. Neither the seat nor the rest alone was prominently linked to LBP [14].

The acceleration values (Ravnik 2002) of some of the vibrations we are exposed to in vehicles are [15]
• car0.20–0.75 m.s^−2^• bus0.40–0.80 m.s^−2^• tractor0.40–2.80 m.s^−2^• forklift0.40–2.00 m.s^−2^• locomotive0.30–0.60 m.s^−2^• tank1.50–3.50 m.s^−2^• ship0.50–0.70 m.s^−2^• helicopter0.10–1.55 m.s^−2^

It is obvious that the dynamic load during the driving of the vehicle results in a load on the crew, which in the long run affects their health. The experiment aimed to determine what vertical acceleration values are transmitted from the vehicle structure to the driver’s and passenger’s body at the highest point of the parietal bone (Vertex Parietal Bone) and the vertebrae of the cervical spine C7 (Vertebrae Cervicales) as the vehicle passes over obstacles at different speeds.

The cervical vertebra C7 was chosen due to the frequency of cervical vertebrae injuries (C0 to C2 = 20%, C3 to C7 = 80%) [1,16,17]. Vertebra C7 is also the most easily palpable.

In the past, researchers have sought to examine the load of the crew when driving in wheeled vehicles in a number of other scientific works that helped in solving this work and writing this article (e.g., Černohlávek 2020, Pelcová et al. 2006; Harrison et al. 2000; Nest 2000; Zhao and Wang 2019; Quoc et al. 2020; Kajiwara et al. 2021; Peng et al. 2020) [18,19,20,21,22,23,24].

As mentioned above, the dynamic loads and response of the vehicle crew are often an issue. This article develops the results of a pilot measurement carried out in 2019 [25]. An earlier pilot measurement proved the suitability of the set measuring chain as well as the suitability of the used acceleration sensors for measuring the dynamic load of the vehicle crew. This article develops the mentioned pilot measurement and gives clearer information about the increase of the dynamic load of the crew when crossing selected inequalities at speeds up to 50 km.h^−1^. The results of this measurement will continue to be used for the construction and verification of a mathematical model in the MSC ADAMS program, where acceleration values at higher speeds will be determined, and which cannot be investigated experimentally due to the risk of crew injury.

## 2. Experimental Solution

**Figure 1:**
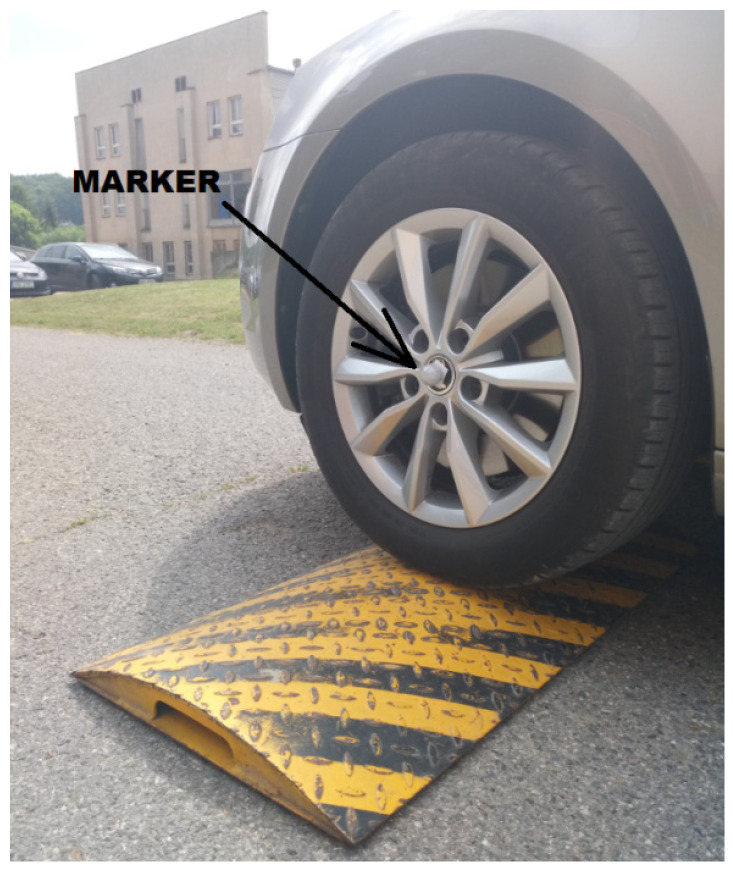
Obstacle on the road.

The measurement was performed experimentally in a Škoda Octavia passenger car (2016). The ride was made on a flat road on which obstacles were placed. The vehicle was excited by passing over 50-mm-high retarders (Figure 1).

**Figure 2:**
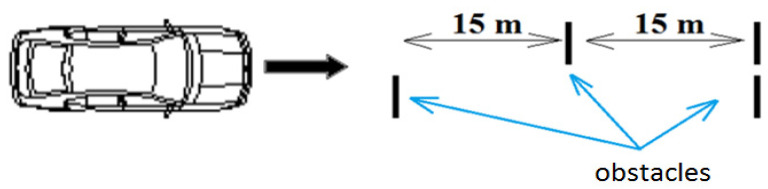
Schematic distribution of obstacles on the road.

Figure 2 shows the distribution of obstacles (cylindrical retarders) on the track. The obstacles were 15 m apart (a sufficient distance to dampen the vehicle’s vibrations). Five obstacle speeds were selected, and the readings were measured in the first five rides on the driver, and then on the passenger (human). The measurement was performed in a total of ten different variants in the number of seven replicates, see Table 1.

**Figure 3:**
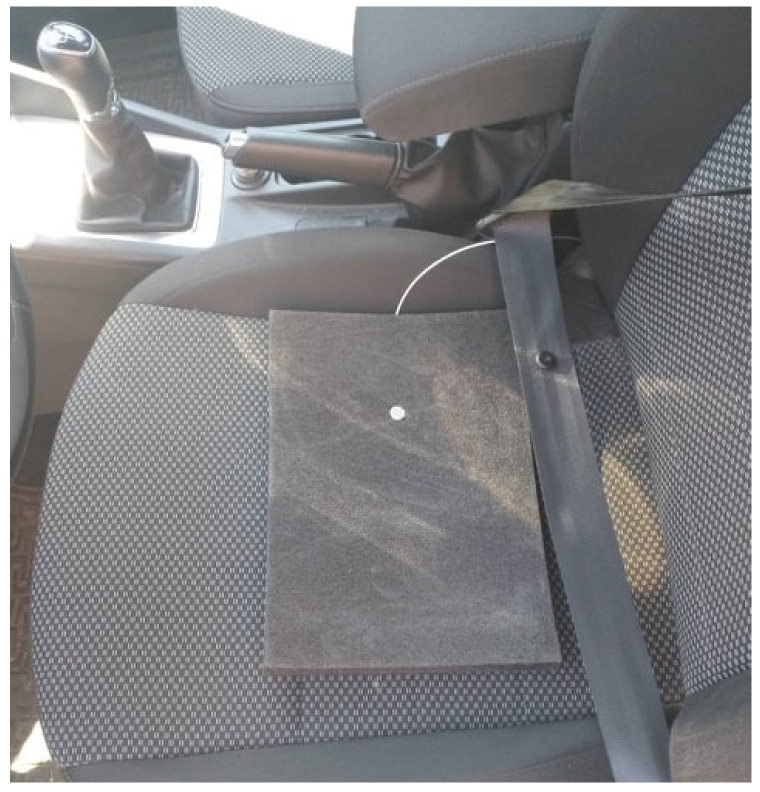
Acceleration sensor placed on the seat of the vehicle under the driver’s buttock.

**Figure 4:**
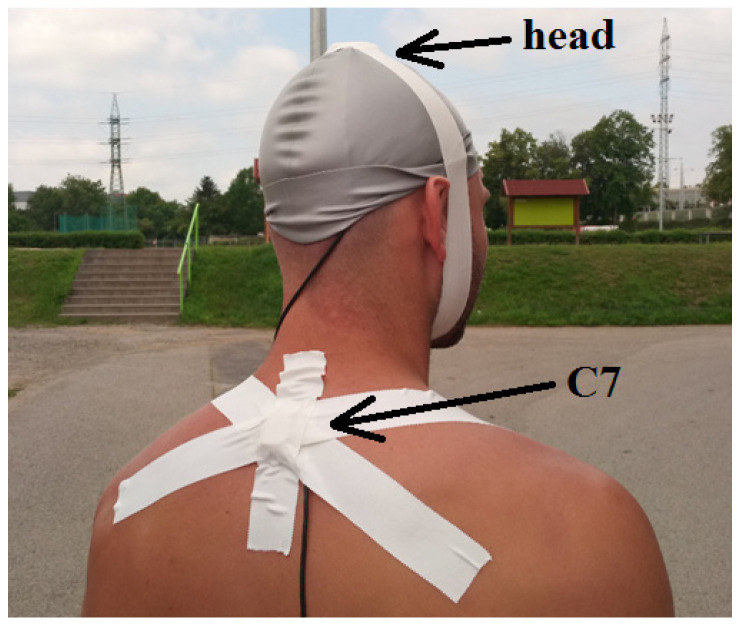
Acceleration sensor placed on the test subject’s head and C7 vertebrae.

The magnitudes of the accelerations generated on the structure of the passenger seat, under the buttocks (Figure 3) at the highest point of the parietal bone of the head (Vertex Parietal Bone) and the cervical vertebra C7 (Vertebra Cervicales), shown in Figure 4, were measured.

**Figure 5:**
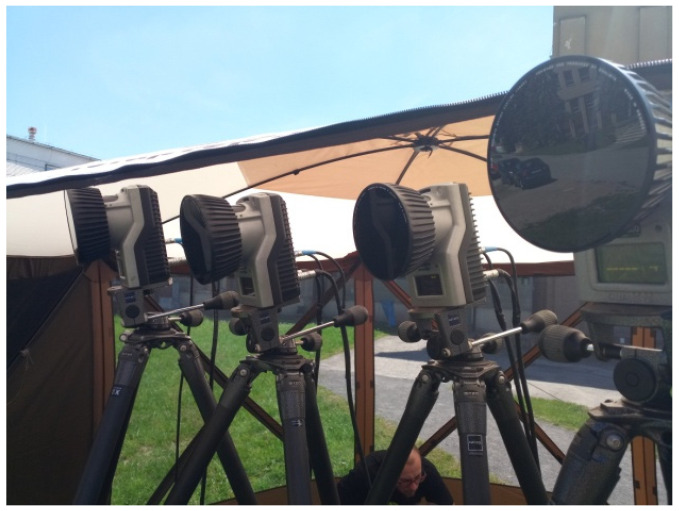
Device for measuring the exact speed of the vehicle—System Qualisys.

**Figure 6:**
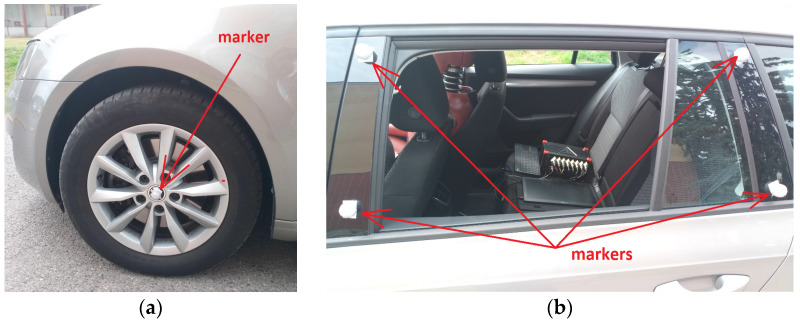
Markers placed on the vehicle: (**a**) wheel of the vehicle and (**b**) body of the vehicle.

The subjects were healthy men (5 men aged from 25 to 30) without injuries. The aim of the work was to determine how much dynamic effects are transmitted from the vehicle structure to the body of the driver/passenger—respectively, to the head and vertebra of the cervical spine C7 (vertebrae cervicales)—when the vehicle is overcoming obstacles. The vehicle speed was also measured by a Qualisys camera system (Figure 5). Using four infrared cameras, the movement of the markers (balls with a diameter of 9 mm) glued to the vehicle (wheels, body) was recorded. A total of nine markers were placed on the vehicle. The marker which was placed on the center of the wheel, can be seen in Figure 1 and Figure 6a,b.

Acceleration and position sensors were connected to MMF preamplifiers: Dewetron MSI-BR-ACC-S1 and SMS: Dewetron MSI-BR-CH-50, on each channel to the Dewesoft STGM + amplifier. The data processing device Dewesoft Siruis Sbox + battery pack was used for signal processing (the device will be placed in a car-scanning frequency of 20,000 Hz, scanned without the use of built-in HW signal filters).

Sensor types:

- 3x triaxial accelerometer MMF KS 943B.100 (10 mV/m.s^−2^)—placed on the head and vertebra C7;
- 1x triaxial accelerometer MMF KS 943B.10 (1 mV/m.s^−2^)—located on the car seat frame under the seat;
- 2x uniaxial accelerometer SMS MH 118 (1 pC/m.s^−2^)—located between the buttocks and the seat and on the wheel suspension.

## 3. Measurements Results

Table 2 shows the average values of the obstacle acceleration measured on the driver (human) of a passenger car vehicle (average acceleration values for measurement variants V1 to V35). Table 3 shows the average values of the obstacle acceleration measured on the passenger (human) of a passenger car (measurement variants V36 to V70).

The sample standard deviation with the acceleration values of the given sensor for the given speed crossing the inequality was calculated according to the formula:

(1) $$s=\sqrt{s^{2}}=\sqrt{\frac{1}{N}\overset{n}{\underset{i=1}{\sum }}{\left(x_{i}-\bar{x}\right)}^{2}}$$

The variance of *Var* () values was calculated according to the relation:

(2) $$Var\left(X\right)=\frac{1}{N}\overset{n}{\underset{i=1}{\sum }}{\left(x_{i}-\bar{x}\right)}^{2}$$

Table 4 shows the measured and processed acceleration values for variants V50 to V56.

The results of the performed measurements were graphically processed in the program Dewesoft X3 and MS Excel.

**Figure 7:**
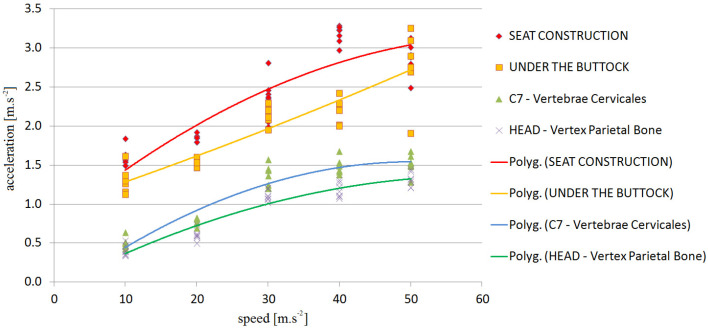
DRIVER-dependence of vehicle speed on acceleration—V1 to V35.

**Figure 8:**
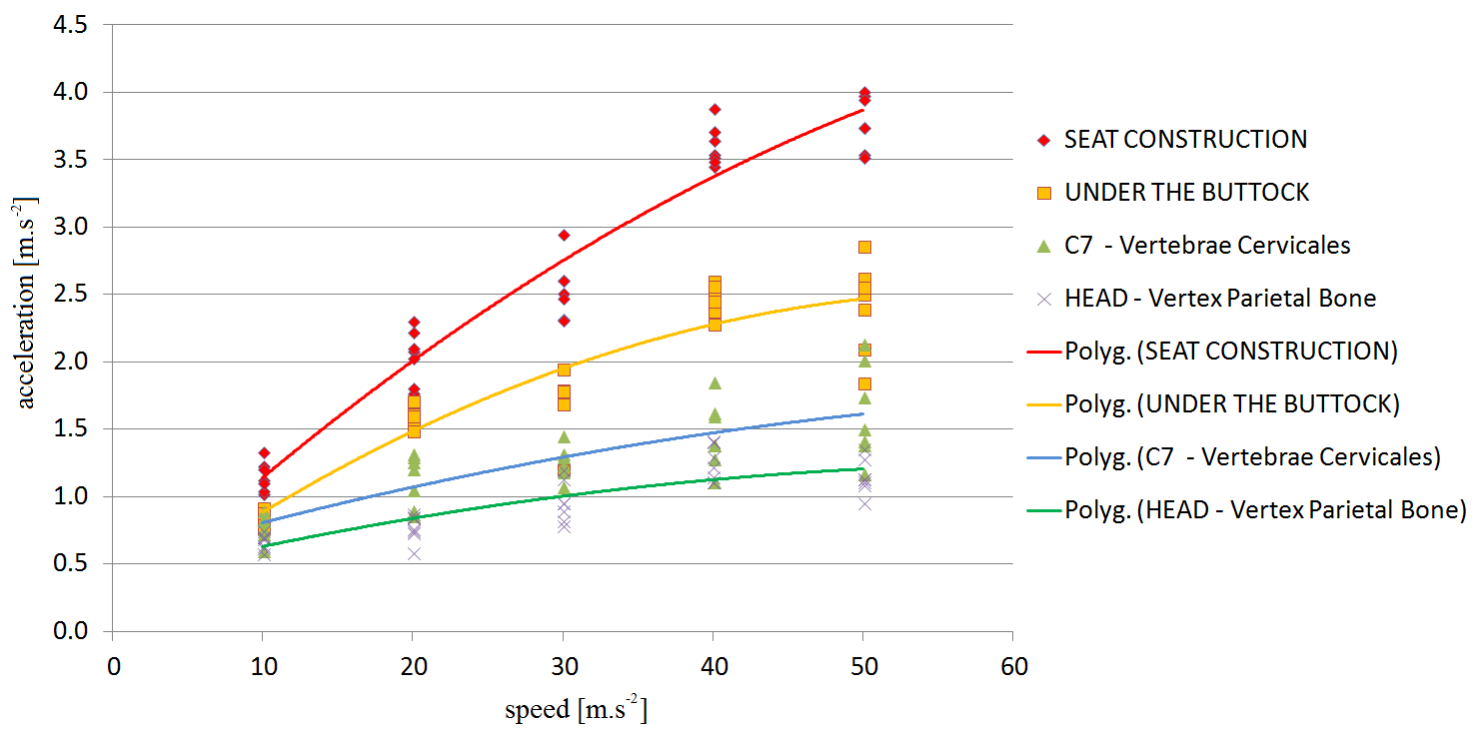
CO-DRIVER-dependence of vehicle speed on acceleration—V36 to V70.

The graphical results are shown in Figure 7 and Figure 8. A set of trends—a polynomial of the second degree—was drawn with a set of measured values to obtain the dependence of the acceleration on the speed at which the obstacles were crossed.

**Figure 9:**
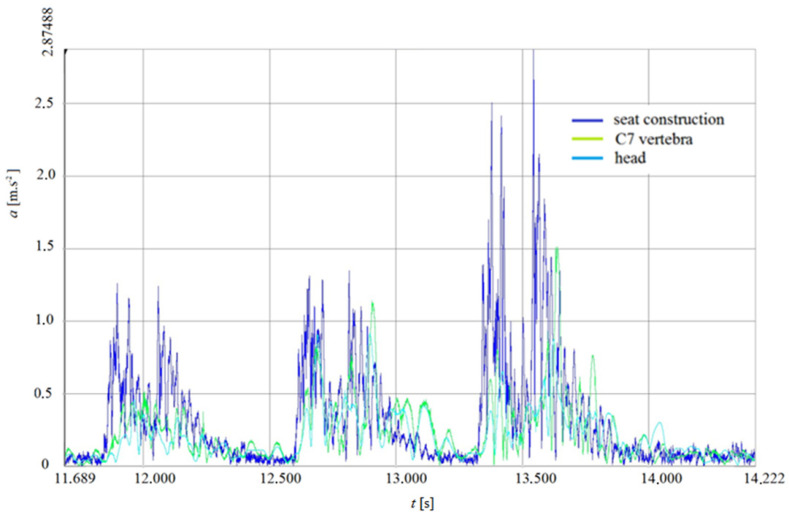
DRIVER (HUMAN)-dependence of acceleration in time of crossing obstacles—V5.

**Figure 10:**
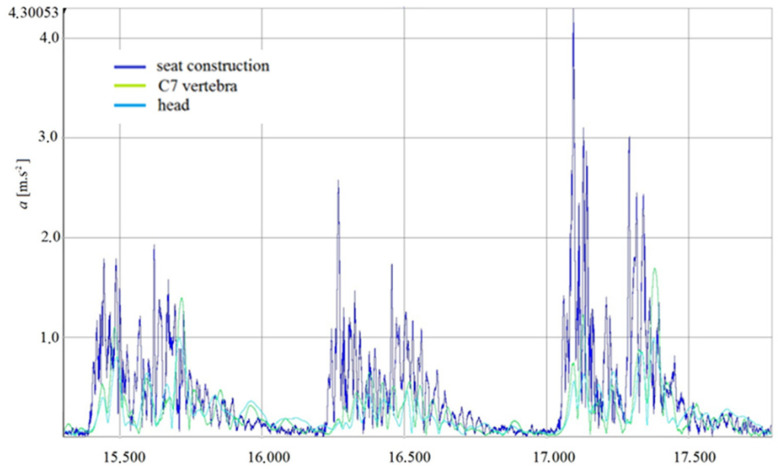
CO-DRIVER (HUMAN)-dependence of acceleration in time of crossing obstacles—V10.

The graph created in the Dewesoft X3 software can be seen in Figure 9 and Figure 10. The graph shows the crossing of all three retarders. The left, the middle and the right part of the graph refer to the right wheel, the left wheel and the acceleration value of both wheels while crossing over the retarders, respectively.

## 4. Conclusions

From the performed measurements, the magnitudes of the average accelerations that affect the vehicle crew while driving in a passenger car when crossing obstacles while driving directly on the road were determined. When crossing obstacles at the highest speed of the vehicle (50 km.h−^1^), an acceleration of 1.516 m.s^−2^ and the highest head peak of 1.316 m.s^−2^ were measured on a person seated in the driver’s seat on the C7 vertebra.

The passenger (human) was also affected by the greatest acceleration when crossing the vehicle at the highest obstacle course speed (50 km.h^−1^). The maximum acceleration of the head was measured at 1.153 m.s^−2^, whereas in the area of the vertebra C7 it was 1.624 m.s^−2^. The measured maximum values on the driver’s /passenger’s body were determined when crossing an obstacle with both wheels at the same time.

Based on the performed measurements, we can state that the driver and front passenger can be burdened with greater dynamic effects in the vehicle than stated in scientific publications; therefore, it is necessary to further discuss this issue and address increasing driving safety for the vehicle crew.

Structural modifications of the vehicle (e.g., attaching a car seat, shaping a car seat or searching for new materials with a higher damping effect) might be a solution

The results of the experiment showed the magnitudes of the dynamic load of the car crew when crossing uneven surfaces at different crossing speeds. The dynamic effects affecting the car crew were measured at the driver’s and front passenger’s seats. It has been shown that a person is burdened by higher acceleration values when driving a car than stated by Ravnik (2005).

The results of our work confirmed previously performed studies that dealt with the dynamic load of people in vehicles [5,10,19,25,26]. This issue needs to be further addressed. Reducing the dynamic load on a vehicle crew can be achieved by further improving the quality of the suspension system. Another possibility to reduce the dynamic load is the possibility of a design modifying the seat attachment, with the possibility of an additional safe suspension mechanism.

## Figures and Tables

**Table 1:** Variants of the measurements.

Speed	10 km.h^−1^	20 km.h^−1^	30 km.h^−1^	40 km.h^−1^	50 km.h^−1^
Driver-human	V1–V7	V8–V14	V15–V21	V22–V28	V29–V35
Co-driver-human	V36–V42	V43–V49	V50–V56	V57–V63	V64–V70

**Table 2:** Average values of accelerations for driver-variants V1 to V35.

Velocity [km.hod^−1^]	Measured Acceleration Values			
	Seat Construction [m.s^−2^]	Under the Buttock [m.s^−2^]	C7 [m.s^−2^]	Head [m.s^−2^]
10	1.556	1.304	0.499	0.407
20	1.805	1.514	0.775	0.604
30	2.350	2.147	1.351	1.109
40	3.178	2.199	1.490	1.179
50	2.875	2.755	1.516	1.316

**Table 3:** Average values of accelerations for co-driver-variants V36 to V70.

Velocity [km.hod^−1^]	Measured Acceleration Values			
	Seat Construction [m.s^−2^]	Under the Buttock [m.s^−2^]	C7 [m.s^−2^]	Head [m.s^−2^]
10	1.156	0.853	0.784	0.671
20	2.049	1.633	1.131	0.772
30	2.542	1.695	1.255	0.965
40	3.608	2.471	1.465	1.245
50	3.786	2.413	1.624	1.153

**Table 4:** Statistical processing of acceleration values-variants V50 to V56 (C7 and HEAD).

Velocity [km.hod^−1^]	C7		HEAD	
	Max. Acceleration [m.s^−2^]	Standard Deviation	Max. Acceleration [m.s^−2^]	Standard Deviation
30	1.201	0.232	1.199	0.016
30	0.786	0.183	0.825	0.150
30	0.955	0.014	1.131	0.371
30	0.922	0.047	0.953	0.028
30	0.839	0.032	0.788	0.262
30	1.080	0.111	0.958	0.176
30	1.001	0.130	0.899	0.241
Average value	0.969	0.017	0.965	0.020
